# Rift Valley Fever Virus: An Overview of the Current Status of Diagnostics

**DOI:** 10.3390/biomedicines12030540

**Published:** 2024-02-28

**Authors:** Daniele Lapa, Silvia Pauciullo, Ida Ricci, Anna Rosa Garbuglia, Fabrizio Maggi, Maria Teresa Scicluna, Silvia Tofani

**Affiliations:** 1 Laboratory of Virology, National Institute for Infectious Diseases “Lazzaro Spallanzani” (IRCCS), 00149 Rome, Italy; daniele.lapa@inmi.it (D.L.); annarosa.garbuglia@inmi.it (A.R.G.); fabrizio.maggi@inmi.it (F.M.); 2 National Reference Center for Equine Diseases, Istituto Zooprofilattico Sperimentale del Lazio e della Toscana “M. Aleandri”, Via Appia Nuova 1411, 00178 Rome, Italy; ida.ricci@izslt.it (I.R.); teresa.scicluna@izslt.it (M.T.S.); silvia.tofani@izslt.it (S.T.)

**Keywords:** Rift Valley fever virus, molecular diagnostics, serology, early diagnosis

## Abstract

Rift Valley fever is a vector-borne zoonotic disease caused by the Rift Valley fever virus (Phlebovirus genus) listed among the eight pathogens included in the Bluepoint list by the WHO. The transmission is mainly vehicled by *Aedes* and *Culex* mosquito species. Symptoms of the disease are varied and non-specific, making clinical diagnosis often challenging, especially in the early stages. Due to the difficulty in distinguishing Rift Valley fever from other viral hemorrhagic fevers, as well as many other diseases that cause fever, an early diagnosis of the infection is important to limit its spread and to provide appropriate care to patients. To date, there is no validated point-of-care diagnostic tool. The virus can only be detected in the blood for a brief period, suggesting that molecular methods alone are not sufficient for case determination. For this, it is preferable to combine both molecular and serological tests. The wide distribution of competent vectors in non-endemic areas, together with global climate change, elicit the spread of RVFV to continents other than Africa, making surveillance activities vital to prevent or to limit the impact of human outbreaks and for a rapid identification of positive cases, making diagnosis a key factor for this achievement.

## 1. Introduction

Rift Valley fever virus (RVFV) is included in the Bluepoint list by the World Health Organization (WHO) together with other pathogens (Ebola virus, Zika virus, Lassa fever virus, Nipah virus, Crimean–Congo hemorrhagic fever virus, severe acute respiratory syndrome coronavirus, and Middle East respiratory syndrome coronavirus), which are prioritized for research and development [1].

RVFV is a vector-borne zoonotic disease caused by a phlebovirus (family *Phenuiviridae*), firstly described in 1931 during an epidemic outbreak in the Rift Valley region of Kenya, with high rates of abortion among pregnant ewes and acute deaths of newborn lambs [2].

**Figure 1:**
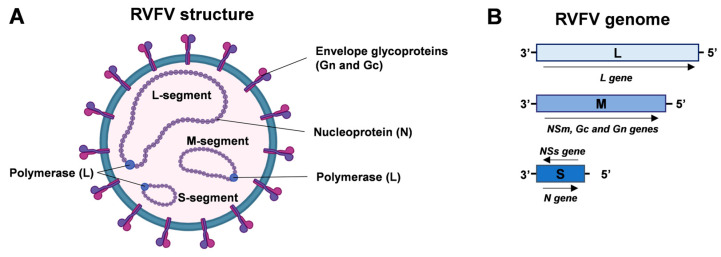
RVFV structure and genome organization. (**A**) Enveloped RVFV virion comprises nucleoprotein (N) and envelope glycoproteins (Gn and Gc). Viral polymerase (L) and N proteins are associated with the viral genomic segments (RNA, negative or ambisense polarity). (**B**) Schematic representation of RVFV genome organization. The L segment encodes the viral RNA polymerase; the M segment encodes NSm, Gc, and Gn proteins; and the S segment encodes the N protein and NSs protein (ambisense polarity). Created with Biorender.com.

The RVFV is a phlebovirus belonging to the order *Bunyavirales* and family *Phenuiviridae.* This virus has a single-stranded RNA genome consisting of three segments: a small (S), medium (M), and large (L) segment, all of negative or ambisense polarity. The S segment encodes for the nucleoprotein (N), while its anti-genomic RNA is responsible for encoding the non-structural NSs protein, which acts as a major virulence factor [3]. The M segment encodes glycoproteins precursor Gc and Gn, together with the nonstructural proteins NSm and the proteins P78, P14, and P13. The L segment encodes the viral RNA-dependent RNA polymerase (RdRp), as shown in Figure 1 [3].

Viral reassortment can occur among Phlebovirus genera due to their segmented genomic RNA. Furthermore, genomic reassortment among different RVFV strains is also known to occur and has been demonstrated experimentally [4].

Ngari virus, which is grouped in the genus Orthobunyavirus within the *Bunyaviridae* family, is a zoonotic arbovirus, and it was identified during a RVFV outbreak in Mauritania in 2010; this can lead to co-infections in goats [5].

Another study with the RVFV MP-12 strain and a genetic variant showed that out of 47 plaques isolated from coinfected C6/36 mosquito cells, 83% were reassortants [6].

Protection against RVFV in all animal species is conferred by neutralizing antibodies, which can be detected within the first week post-infection [7]. Nucleoproteins (N) induce high levels of IgG and IgM antibodies in RVFV and other bunyavirus infections, but there is no evidence that anti-N antibodies exhibit virus-neutralizing activity [8].

In a study, long-lived IgG and T cell responses were detected against viral envelope glycoproteins, Gn and Gc. However, antigen-specific antibody depletion experiments showed that Gn-specific antibodies dominate the RVFV neutralizing antibodies (nAb) response. Finally, IgG avidity against Gn, but not Gc, correlated with nAb titers [9].

Recently, Quellec et al. (2023) showed that RVFV-infected astrocytes upregulated expression of genes associated with inflammatory and type I interferon responses at the mRNA level but not at the protein level [10].

**Figure 2:**
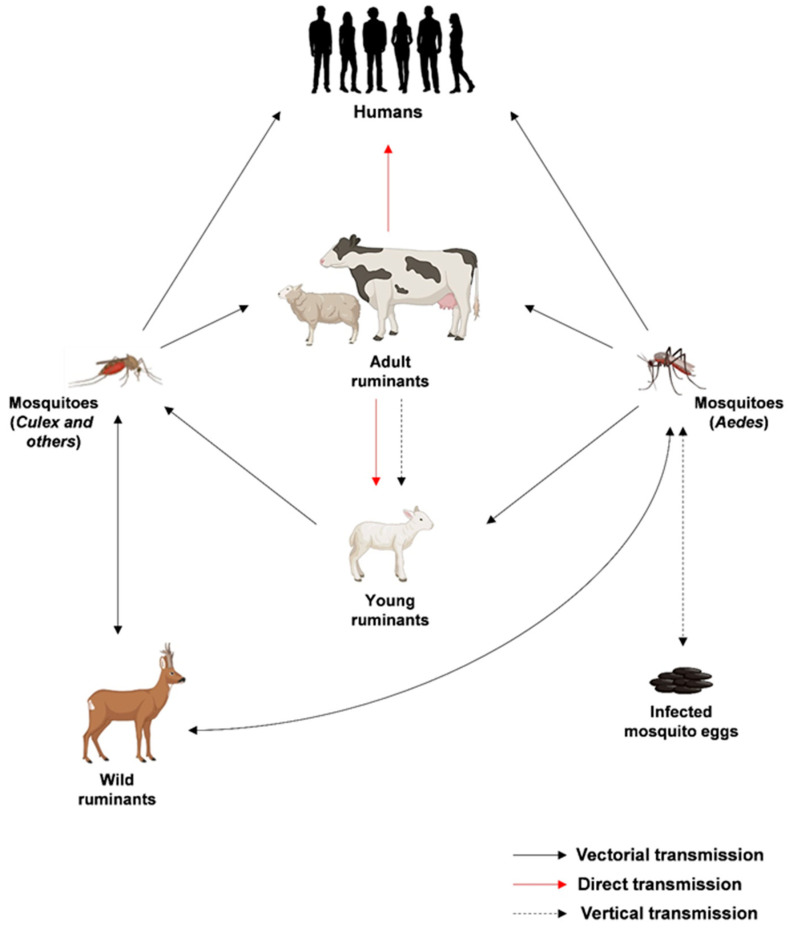
Epidemiological transmission cycles of RVFV. Created with Biorender.com.

The life cycle of RVFV is intricately linked with its transmission dynamics and the interactions between the virus, mosquitoes, and vertebrate hosts. Transmission predominantly occurs through the bites of infected mosquitoes, with *Aedes* and *Culex* species being key vectors. Upon entering a host organism, RVFV targets specific cells, initiating its replication process, which mainly occurs in the cell cytoplasm and probably starts near the site of endosomal fusion [11]. The S, M, and L segments are transcribed and translated, leading to the production of viral proteins and the formation of new viral particles within the Golgi apparatus. The assembled virions are then released from the host cell by exocytosis, allowing the virus to spread within the host organism and causing infections in various tissues. Importantly, infected animals serve as reservoirs for the virus, facilitating its transmission to mosquito vectors during blood feeding. As mosquitoes become infected, they play a crucial role in perpetuating the cycle by transmitting the virus to other animals or humans during subsequent bites. The transmission cycles of RVFV are summarized in Figure 2.

RVFV has an incubation period of 2–6 days in humans.

In most cases, people infected with RVFV are asymptomatic or paucisymptomatic (fever, headache, weakness, back pain, vertigo, anorexia, photophobia, and dizziness). Approximately 10% of people with RVFV develop severe symptoms, with eye disease in 0.5–2% of patients. Encephalitis or inflammation in the brain is observed in 1% of cases. Finally, only 1% of cases may manifest hemorrhagic fever. Hemorrhagic fever can lead to up to 50% fatality, and after about a week after the onset of hemorrhagic symptoms, there is death [8,12].

A histopathological examination of infected human brains demonstrated encephalitis with an infiltration of lymphocytes and necrosis in neurons [13]. *Drosophila melanogaster* (*D. melanogaster*) and vertebrates have similarities for the neural proliferation and brain circuit formation. Therefore, *D. melanogaster* can be used to better understand what cellular and molecular mechanisms of Rift Valley viruses are involved in neurological disorders [14].

Wild animals tend to have mild or inapparent infection, whereas domestic animals are more susceptible to RVF disease [15]. Clinical signs range from sudden death or abortion to mild, non-specific symptoms, depending on the virulence of the virus strain and the species involved. Mortality may reach 70–100% in lambs and kids, and 20–70% in adult sheep and calves. Cattle and camels are less susceptible to infection, and abortion rates may reach 85–100% within the affected herds [16]. In pregnant sheep or goats, RVFV infection results in above 100% fetal mortality [17].

RVFV infection in older non-pregnant animals is often asymptomatic, and abortion may be the only overt manifestation of the disease in a herd or flock [18].

Transmission between animals by direct contact with infected tissues or fluids has been observed, together with iatrogenic route by use of infected needles used for vaccination, particularly in endemic regions with limited economic resources [7,19]. The infections in humans can occur by inhaling aerosols of infectious body fluids, and consumption of raw or unpasteurized milk has also been identified as a risk factor for RVFV infection [12].

The RVFV is transmitted from different mosquito species to animals or humans and from infected animals to humans [8,20].

RVFV-competent mosquito vectors fall under 73 species of mosquitoes in the 8 genera of the family *Culicidae*. Trans-ovarian or vertical transmission is also reported [20,21].

It is transmitted from ruminants to ruminants by mosquito bites, mainly from the genera *Aedes* and *Culex*, but also from the genera *Anopheles* and *Mansonia*, as recently suggested in Madagascar and Kenya [22,23]. Animals are infectious for mosquitoes during the viremic period. Viremia may be brief (6–18 h) or persist for 6 to 8 days [24].

However, the severity of clinical signs is different on the species: sheep are more susceptible than goats, which are themselves more susceptible than cattle and camels [25].

Human-to-human transmission of the RVFV has not been demonstrated, but vertical transmission readily occurs in animals and has also been reported in humans [26,27].

Outbreaks from West African Sahelian areas are not closely related to high rainfall, which is a contributing factor. In years with short-term dry periods punctuated by occasional precipitation, double cycles of *Aedes* vectors are observed, together with simultaneous waves of *Aedes* and *Culex* vectors [28,29].

Most human cases do not require treatment against RVFV. For severe cases of Rift Valley fever, there is no specific treatment but only a general supportive care treatment [12].

Several outbreaks have been described as causing severe economic and health consequences [1].

Overall, from 2000 to 2016, 11 outbreaks of RVFV occurred in humans in the Republic of Niger (2016), the Republic of Mauritania (2012), the Republic of South Africa (2010), Madagascar (2008 and 2009), Sudan (2007), Kenya, Somalia, Tanzania (2006), Egypt (2003), Saudi Arabia, and Yemen (2000) [30]. The fatality rate was about 19.5%, with 950 reported death cases [30]. To date, no outbreaks have been reported in Europe [31].

The percentage of seroprevalence in humans ranges from 1.8% (Kenya) to 11.1% (Saudi Arabia) [32,33]. In countries along the Mediterranean basin, anti-RVFV IgG prevalence ranged from 1.4% in Tunisia to 4.9% in Turkey [34,35], while in Italy, the authors found the antibody anti-IgG Rift Valley fever virus in 2.6% of serum patients with suspect arbovirus infection [36].

Wild animals have been found to be seropositive for RVFV antibodies; during the epizootic period in 2006–2007, the 31.8% of wild ruminants surveyed in Kenya were positive for anti-RVFV antibodies [37].

Considering that mosquitoes of the *Aedes* and *Culex* species are now circulating in Italy and Europe, mostly due to climate change, there is a risk of arbovirus introduction to continents other than Africa [38].

## 2. Epidemiology

The first reported case of RVFV was in 1930 in Kenya, during an outbreak in sheep [30]. Afterwards, outbreaks affecting livestock and humans occurred in other countries of Africa such as Egypt, Kenya, South Africa, Madagascar, Mauritania, Senegal, and Gambia [38]. Over the past 25 years, RVF disease has expanded its historic geographic range in the livestock-raising areas of eastern and southern Africa and into the Middle East (Saudi Arabia and Yemen), following an infected livestock trade from the horn of Africa, causing several epizootics and epidemics [39]. This first episode of RFV in humans and animals outside the African continent raised concerns that it could be extended in other parts of Asia. According to what was reported for 2019 from the CDC, as for 2017 and 2018, European countries reported four cases of RVF in humans. There were imported cases from Western Africa: three from Mali (first confirmed case in 2015 and second European confirmed cases in 2016) [30,40] and one from Ghana (one suspected case in 2016) [41].

**Figure 3:**
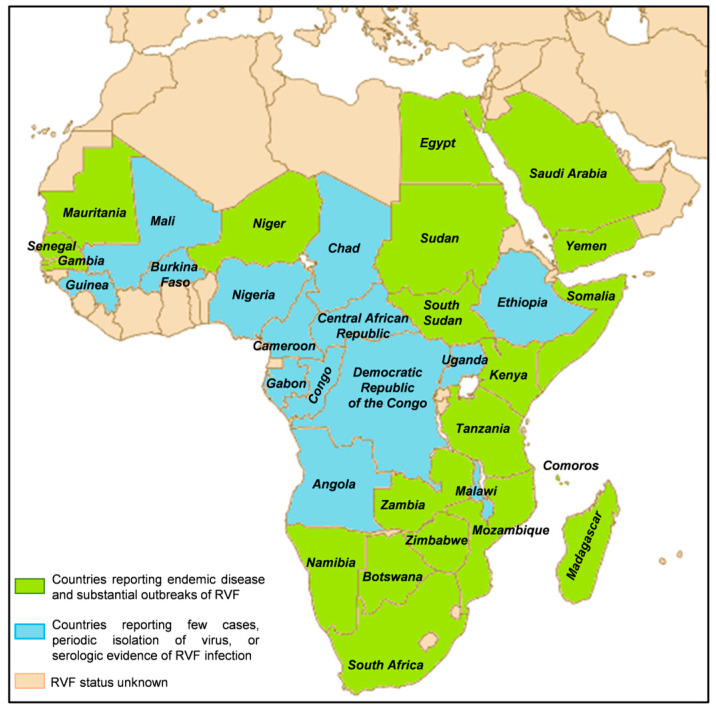
RVF distribution map. Created with Biorender.com.

All cases were males, and the mean age at infection was 32 years. All four cases were European residents. No associated deaths were reported [42]. RVFV was historically confined to the African continent until 2000, and it is enzootic in many African countries and Madagascar, see Figure 3. Global changes, including climatic ones, may cause an expansion in the geographical distribution of the RVFV, together with legal or illegal animal movements and mosquitoe distribution [43,44].

## 3. Diagnosis

The early stages of Rift Valley infection are difficult to diagnose because the symptoms are nonspecific. In particular, the symptomatology is similar to that of other hemorrhagic fevers and fever-causing infections such as malaria, shigellosis, typhoid fever, and yellow fever.

### 3.1. Molecular Diagnostics

The RNA detection of the RVFV genome has been performed over the years with several methods.

At the beginning of the 2000s, a one-step RT-PCR method was developed with a sensitivity of 0.5 pfu/reaction. This method was able to detect several RVFV strains such as Gabek, Forest, Gordil, Saint Floris, Arumwot, Belterra, ArD38661, AnD100286, and MP12 [45]. Afterwards, different real-time PCR methods were developed, targeting the G2 gene, a region of the S segment [46,47,48,49]. RT-loop-mediated amplification (RT-LAMP) is currently the most used platform. This method uses different regions of the viral genome as a template: L segment [50,51], S segment [52,53], or M segment [54]. The most sensitive assay was based on the L region target: 10 copies/reaction [51]. Moreover, an isothermal recombinase polymerase amplification (RPA) assay had been set up by Euler, reaching a low limit detection (LLOD) of 19 RNA molecules/reaction [53]. Furthermore, RVFV detection was included in a multiplex RT-qPCR for hemorrhagic fever pathogens. However, the sensitivity was very low in comparison with those reached by RT-LAMP or real-time RT-PCR (10^5^–10^6^ copies/mL) [55]. The identification of different RVFV strains could be carried out by analysis of the melting curve of the L, M, and S regions amplification [56]. Other assays that were based on the microarray method were developed; one detecting the GP gene was validated with a real-time PCR, but few samples were tested, and no data about specificity were reported [57]. A second one detected the M segment with an average detection limit of 6.36 DNA copies, targeting the other 28 target pathogens on the array in addition to the RVFV, see Table 1 [58].

Although NGS (next-generation sequencing) has been applied for the detection of numerous arboviruses, including Chikungunya, Zika [59], and West Nile viruses [60], and show a sensitivity comparable to real-time PCR, it has not yet been developed for RVFV.

**Table 1:** Molecular assays for RVFV.

Test	Test Type	Virus Detected	Target Gene	Biological Matrix	Reference Assay	Sample Size	Sensitivity	Specificity	Author
RVFV RT-nested PCR	Homemade one-step RT-PCR nested method	RVFV different strains (Gabek forest, Gordil, saint Floris, Arumwot, Belterra, ArD38661, AnD100286, MP 12)	NS coding region of S segment	Virus produced in Vero E6 cells; serum from infected mice	Virus isolation method	ND	0.5 pfu/reaction	Nd	Sall et al., 2001 [45]
RVFV quantitative real-time PCR	qRT-PCR with fluorescent signal from probes for quality control	RVFV; MP12, ZH501, ZH548, ArD38661, 74 HB59 strains	NS coding region of S segment	Virus produced in Vero E6 cells; serum from infected mice	ND	ND	50–100 copies/reaction	No amplification with Toscana, Icoraci, and Belterra closely related phlebovirus	Garcia et al., 2001 [46]
RT-Real-time PCR	5′ nuclease technology on a light cycler instrument	RVFV	G2 gene	Synthetic RNA	ND	ND	2835 geq/mL	no cross-reactivity with other HCV, HBV, HSV1, CMV, *Modoc virus*, *Mycobacterium tuberculosis*, *Mycobacterium leprae*, *Borrelia* spp., *Leptospira* spp., *Neisseria* spp., *Plasmodium* spp., *Leishmania* spp.	Drosten et al., 2002 [48]
RT-Real-time PCR homemade	Fluorescent nested PCR TaqMan assay	RVFV	S segment	Synthetic RNA	ND	ND	100 copies/reaction	SFNV cell culture	Weidmann et al., 2008 [47]
Real-time qRT-PCR homemade	qRT-PCR with fluorescent reporter dye detected at each PCR cycle	RVFV	G2 gene	Plasma of suspected patients with HVF	272 RVFV confirmed cases	2ND	100 infectious particles/mL	IgM anti RVFV positive sera 100%	Njenga et al., 2009 [49]
RT-LAMP homemade	Reverse transcription-loop-mediated isothermal amplification with a vertical	RVFV	L segment	Serum samples	TaqMan Real Time	64	Whole blood: LLOD: 10 copies RNA/reaction	No cross reactivity with phleboviruses; flaviviruses and chikungunja virus	Peyrefitte et al., 2008 [50]
RT-LAMP homemade	Reverse transcription-loop-mediated isothermal amplification with a vertical	RVFV	L segment	Bleed samples from sheep (n = 20), human plasma from suspected cases (n = 65); 3 liver, kidney, serum from animals			Whole blood: LLOD: 10 copies/ reaction	Six African phleboviruses and unrelated arbovirus did not give cross reactivity	Le Roux et al., 2009 [51]
RT-LAMP homemade	Reverse transcription-loop-mediated isothermal amplification	RVFV	S segment	Synthetic RNA	Real-time RT-PCR	ND	whole blood: LLOD: 1.94 copies/microliters within 60 min	No cross reactivity with JEV, H3N2 influenza virus, EBOV, MARV	Han et al., 2020 [52]
RT-LAMP homemade	Reverse transcription-loop-mediated isothermal amplification	RVFV	M segment	Blood samples	Real-time RT-PCR	130	98.36% sensitivity	100%; no cross-reactivity with PPR and capripox viruses	Wekesa et al., 2023 [54]
Isothermal recombinase polymerase amplification (RPA)	Isothermal exponential nucleic acid amplification and detection method	RVFV	S segment	Synthetic RNA	ND	ND	19RNA molecules/reaction	No cross reactivity with Yersinia pestis, Francisella tularensis, Bacillus antracis, vaccinia virus, Ebola virus, Marburg virus, Crimean–Congo virus and phleboviruses	Euler et al., 2012 [53]
RT-qPCR genotyping assay	One step RT-qPCR for typing different strains of RVFV, melting curve to identify different strains of RVFV	RVFV	L, M, S segments	ND	Sanger sequencing		ND		Balaraman et al., 2023 [56]
BioT DNA multiplex PCR-enzyme hybridization assay	Multiplex RT-PCR	RVFV	GP2 gene	196 swabs, 45 skin swabs,15 serum, 7 sputum	ND	260 clinical samples	10^5^–10^6^ copies/mL with nucleic acid extraction	No cross reactivity with Influenza A, EBV, CMV, RSV A, ADV C, human metapneumovirus	He et al., 2009 [55]
Oligonucleotide microarray	Microarray	RVFV	GP gene	Culture samples	Real-time PCR	60	100%		Yao et al., 2021 [57]
Real-time qRT-PCR commercial	qRT-PCR with fluorescent reporter dye detected at each PCR cycle	RVFV					0.89 copies/μL	cross-reactivity with flavivirus, Marburg virus, and Ebola virus	[61]

ADV, adenovirus; CMV, cytomegalovirus; RSV, respiratory syncytial virus; JEV, Japanese encephalitis virus.

### 3.2. Serological Diagnosis

Serological assays are key to epidemiological studies for the identification of active infection or previous exposure to the RVFV [62]. Serological diagnosis of RVF can be performed in the laboratory using the following tests:

- Virus neutralization test (VNT)
- Indirect immunofluorescent assay (IFA)
- IgG and IgM antibody enzyme-linked immunosorbent assay (ELISA)

ELISA tests and virus neutralization tests are the most widely used methods for antibody detection. The agar gel immunodiffusion (AGID), radioimmunoassays, hemagglutination inhibition (HI), and complement fixation (CF) are disused. Animal samples used for antibody detection may contain live viruses, and therefore, it would be important to apply inactivation procedures before proceeding to the sample testing. The inactivation step could be conducted by a combination of thermal and chemical inactivation [63].

Multiplex detection systems for the detection of antibodies against multiple highly pathogenic agents simultaneously are being developed, making them valuable tools for disease surveillance and diagnosis [64,65]. In the case of RVFV, a specific in situ hybridization (ISH) has been reported for the detection of viral RNA of several RVFV strains in different fixed tissues [66].

#### 3.2.1. Virus Neutralization Test Assay

To detect specific neutralizing antibodies against RVFV, neutralization tests to the virus are used, such as the plaque reduction neutralization test (PRNT).

**Figure 4:**
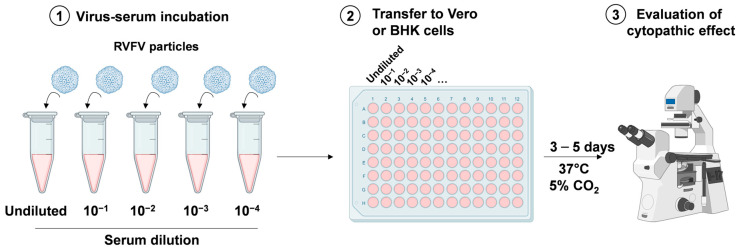
Viral neutralization assay. Serum to be tested is serially diluted and incubated with a defined quantity of viral particles (1). The virus-serum solutions are added to Vero or BHK cells (2) and incubated for 3/5 days at 37 °C in a humidified atmosphere with 5% CO_2_. Finally, the cytopathic effect of viral infection is evaluated through microscopic observation (3). Created with Biorender.com.

VNT is generally accepted as one of the standard assay systems for the quantitative determination of neutralizing antibodies in serum samples in naturally infected and vaccinated animals, but while it is highly specific and useful to test samples from any species, it is also costly, time consuming, and requires a high biosecurity laboratory capable of working safely with live RVFV. VNT is very specific, with cross reactions with other phleboviruses being limited [67,68]. This assay is performed in a BSL3 laboratory and requires highly specialized and vaccinated personnel. VNT is useful to check the protective immunity of a serum that is correlated to the level of neutralizing antibodies, see Figure 4. The sera are serially diluted and incubated with a well-defined number of viral particles (100–300 median tissue culture infective dose (TCID_50_) per mL) before being added to Vero or baby hamster kidney (BHK) cells (3–4 × 10^5^ cells per mL). After 3/5 days, the cytopathic effect of RVFV infection can be observed using a microscope. For confirmation of the results, plates can be fixed with 10% formalin containing 0.05% crystal violet and re-visualized [69]. Gn and Gc are targets for neutralizing antibodies and play an important role in host cell entry and exit [70].

#### 3.2.2. Viral Isolation

RVFV can be identified during the acute phase of the disease by viral isolation from whole blood or serum. In post-mortem cases, the virus can also be isolated from other body districts such as the brain, liver, spleen, and organs of aborted fetuses [71].

In natural and experimental infections, RVFV infects many tissues.

**Figure 5:**
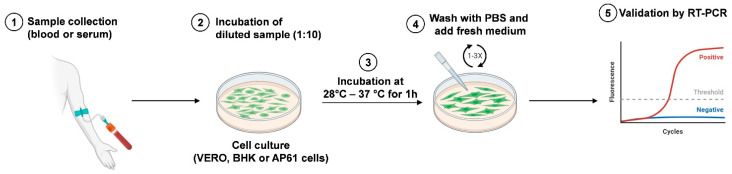
Schematic representation of RVFV isolation. The blood or serum sample is collected from infected hosts (1) and inoculated in cell culture (2). After incubation at 28 °C (AP61 cells) or 37 °C (Vero and BHK cells) for 1 h (3), cell monolayers are washed with PBS and fresh medium is added (4). Virus isolation is validated by RT-PCR analysis (5). Created with Biorender.com.

The RVFV antigen can be present in various tissues of naturally infected sheep, such as the liver, kidney, lungs, testes, vascular tissues, and adrenal glands [72]. In the past, an in vitro culture of live agents was considered the principal method for viral detection, but it has been replaced by PCR and next-generation sequencing. This assay is not used for primary diagnosis because it is less sensitive than PCR. Rift Valley viral isolation, Figure 5, can be performed in different cell lines, including African green monkey kidney (Vero), baby hamster kidney (BHK), and AP61 mosquito cells [73]; a period ranging from 3 to 6 days is necessary to detect the cytopathic effect, and the procedure requires a level 3 laboratory (BSL3 facilities).

They are inoculated with 1/10 dilution of the sample and incubated at 37 °C for 1 h (in the case of mosquito cell lines, the incubation should be performed at 28 °C for 1 h).

After removal of the inoculum, washing with phosphate-buffered saline (PBS) or culture media is performed. Finally, the wash solution is removed, and culture medium is added and incubated for 5–6 days.

The confirmation of virus isolation should be performed using a reverse-transcription polymerase chain reaction (RT-PCR) [74].

#### 3.2.3. Indirect Immunofluorescent Assay

Immunofluorescence assays are still used, although cross-reactions may occur between RVFV and other phleboviruses [69].

An indirect immunofluorescent assay (IFA) using virus-infected Vero cells can be used for the detection of antibodies of Rift Valley fever viruses in BSL3. The samples can be tested at a screening dilution 1:20, both for IgM and IgG detection. Moreover, the positive samples were serially diluted from 1:20 down to 1:1280 to estimate the antibody titer. The IgG and IgM titers are reported as the reciprocal of the highest dilution with positive fluorescence [36]. To date, there are Euroimmun commercial tests for IFA Rift Valley fever virus IgG and IgM detection [75].

#### 3.2.4. ELISA Assay

The ELISA test is a sensitive and rapid test suited to the needs of large-scale testing and the most widely used method for the detection of IgM and IgG isotypes, also commercially available.

IgG and IgM are indeed key markers for RVFV seroprevalence studies, but data on kinetics or persistence during human convalescence are limited. ELISA tests are used routinely in many countries for single-case diagnosis, outbreak management, and surveillance. IgM-capture ELISA allows for the diagnosis of recent infections. IgG-ELISA could be used to determine the rise in antibody response.

The ELISA test is a very versatile test that can be performed with an inactivated antigen, but cross-reactions may occur between the RVF virus and other phleboviruses.

An ELISA (enzyme-linked immunosorbent assay) kit is commercially available to detect anti-Rift Valley fever virus IgG and IgM antibodies, and it is used for serological diagnosis in ruminant animals. At the earliest, it can detect antibodies as soon as four days following infection or vaccination in animals reacting very early, and eight days post-vaccination for 100% of animals. The diagnostic sensitivity was in cattle 84.31%, buffalo 94.44%, sheep 98.91%, and goat 99.18%. The diagnostic specificity was in cattle 99.34%, buffalo 98.28%, sheep 99.16%, goat 99.23%, and other game ruminants 99.26% [76]. Another indirect ELISA based on a recombinant RVFV nucleoprotein has been developed using the cloning, sequencing, and bacterial expression of the N protein of the RVFV. Sera from 106 laboratory workers vaccinated with inactivated RVFV, 16 patients infected with RVFV, 168 serial bleeds from 8 sheep infected by experimentation with RVFV, and 210 serial bleeds from 10 sheep vaccinated with a live attenuated Smithburn RVFV strain were used in this study. The sera positive in the indirect test (I-ELISA) were also positive in the virus-neutralization test. A high correlation (R^2^ = 0.8571) was found between the tests in human vaccines [77].

Another study also used the recombinant nucleoprotein (N) of the RVF virus to test for diagnostic applicability in an indirect ELISA (I-ELISA). The experimentally infected sheep were 128, vaccinated sheep were 240, and field-collected sera in sheep were 251, goats were 362, and cattle were 100. In goats, the diagnostic sensitivity and specificity of the I-ELISA was 100% when using the anti-species IgG conjugate. Using protein G as a detection system, the sensitivity and specificity in goats were 99.4% and 99.5%, respectively; in sheep, the field sera were both 100%, while in cattle, they were 100% and 98.3%, respectively. The I-ELISA based on the recombinant N-protein has the potential to complement the traditional assays for the serodiagnosis of RVFV [78].

An inhibition (competitive) ELISA has been validated, and the sensitivity and specificity were valuated. In fact, it is now commercially available for the detection of antibodies in different species and in domestic and wild ruminants. Field samples were collected from mainland France for the known-negative sera in 192 cattle, 119 goats, 192 sheep, and from ruminants of a French overseas territory (Mayotte) for the known-positive sera. This study showed a sensitivity and specificity of 100%. The results demonstrate that this ELISA may be a suitable diagnostic tool for disease surveillance programs [79].

Commercial assay kits are available, and several in-house protocols have been published [80,81,82]; furthermore, any ELISA has been validated for the detection of antibodies in different species, including humans and domestic and wild ruminants [79,83]. Sensitivity and specificity vary according to the different antigens employed and species under investigation [83,84].

## 4. Surveillance in Humans and Animals

According to the Commission Implementing Regulation (EU) 2018/1882, RVF is categorized as a Category A disease [85] and a notifiable disease, as it has been designated by the World Organisation for Animal Health (WOAH). To date, there is no EU legislation or diagnostic manual for the early detection or surveillance with respect to RVF (EFSA). Except for the outbreaks involving multiple human cases in a French overseas department (Mayotte) in 2018–2019, RVF has never been reported in continental Europe or in countries neighboring the European Union.

According to EFSA opinion [86], because of the possible risk of introduction of RVFV into the EU through vector imports (ports, airports, cargo, and container yards), cattle and small ruminant herds should be included in a surveillance system.

Specifically, passive surveillance can be considered as a first choice for early detection of infection during the peak and late vector season. This should be conducted in areas with the highest risk of introduction (EFSA) in cases of reported animal abortions, stillbirths, or neonatal mortality of cattle, sheep, and goats.

Infected hosts can function as the initial amplifying host, but importation of animals is under restrictions of international trade and checked by the veterinary system. RVFV can be spread to a new geographic area via the movement of infected vectors that can be dispersed via wind, with an estimated travel distance ranging from 110 to 1350 km within 24 h [87], or mechanical transport. This last hypothesis is more likely to be the potential mode; numerous vectors have been discovered alive within aircraft and luggage after international flights [88]. In Kenya, an enhanced surveillance for RFV has been performed, preventing a possible spillover to humans, through a pilot model communication network for emergency reporting of animal health status between farmers, county government surveillance officers, and the national government [89]. Due to climate change, together with the globalization of the animal trade and the wide variety of mosquito species, vector surveillance is crucially important in detecting virus activity as early as possible for a rapid response to reduce an outbreak [90]. Using a model called MINTRISK (Method to INTegrate all relevant RISK aspects), the risk of the introduction of RVFV in the EU was estimated to be very low in all regions (less than one outbreak every 500 years) by the movements of both infected animals and/or vectors [38], but it is not excluded that animal movement may contribute to viral spread, threatening countries in Europe where competent vectors are present [43].

Nevertheless, the surveillance of EU countries should be strengthened due to the spread of the virus in the neighboring countries.

## 5. Discussion

To date, there is no validated point-of-care diagnostic tool. Rapid point-of-care tests would be critically important because they can be used at the patient’s bedside and would provide an alternative that does not require handling with clinical specimens and extraction of nucleic acids from samples. These rapid tests in this way should be easy to use but compatible with biosafety, inexpensive, and do not require well-trained personnel, making diagnosis faster.

Classical diagnosis employs mainly expensive and not so easy-to-use RT-PCR assays, which must be implemented on specific and expensive machines by personnel trained for this purpose, and there are few laboratories capable of performing serosurveillance in asymptomatic people [38,91].

There are few BSL-3 reference laboratories where samples can be managed, not always in the country of the epidemic. The fact that the virus can only be detected in the blood for a brief period (3–5 days post onset of the disease) suggests that RT-PCR alone is not sufficient for case determination. It is preferable to combine both RT-PCR and IgM ELISA or IFA (IgM has a 6-week window in the blood). However, the quality of in-house tests is frequently unknown, and there are few validated serology assays for human specimens. It is necessary to investigate the development of diagnostics intended to detect viruses in other body fluids. Before beginning vaccine clinical trials, laboratory capacity and surveillance systems must be strengthened, and diagnostic tests must be evaluated and validated. For test evaluation, having access to well-characterized samples and reference standards will be necessary (WHO, efficacy trials of Rift Valley fever vaccines and therapeutics) [30]. In 2019, a project (VHFMoDRAD) involving 13 partners started, with an end date set for 31 December 2023. The aim was to develop and deliver rapid and multiplex point-of-care diagnostic tools that will significantly increase the capacity to handle outbreaks of filoviruses and other viral hemorrhagic fever diseases in Africa, such as RVFV. In this project, Gregor Km et al. (2021) carried out a study where they observed how different antibodies can be useful for diagnosis and detection of RVFV in tissues from animals or mosquitoes. The authors found that antibodies against nucleoproteins can be a good tool to find infected cells in animals such as sheep and mice, and antibodies against glycoproteins can be used to find infected tissues in insects [92]. 

Finally, the development of multiplex detection systems serves numerous purposes, such as surveillance, monitoring the presence of RVFV in animals or humans, diagnosis, confirming RVFV infection, and research, studying the biology of RVFV and developing new vaccines and treatments. Despite some limitations, such as complexity, elevated cost and training requirements, compared to traditional diagnostic methods, multiplex detection systems offer several advantages: sensitivity, detecting smaller amounts of viral antigens; specificity, distinguishing between different pathogens; and rapidity, delivering results relatively quickly [64,65].

These findings bring us one step closer to the development of effective diagnostic tools.

## 6. Conclusions

Since 2019, the WHO has added RVFV to the Blueprint priority disease list. Surveillance activities and the rapid identification of positive cases are still important to prevent or limit the impact of human outbreaks, and diagnosis is a key factor for this success.

A plausible pathway for the introduction of RVFV in Europe, in particular for those countries where this can occur, is the movement of infected animals and vectors (shipped by air, sea container, or road transport) [38]. To mitigate the efforts in the risk areas, besides enhanced surveillance activities, there can be the imposition of animal movement restrictions/quarantines, the distribution of mosquito nets, and the dissemination of information to reduce human contact with infected animal products and vectors [91].

## Data Availability

Not applicable.
